# Physiological Responses of Laying Hens to Chronic Cold Stress and Ammonia Exposure: Implications for Environmental Management and Poultry Welfare

**DOI:** 10.3390/ani15121769

**Published:** 2025-06-16

**Authors:** Dapeng Li, Fuwei Li, Wei Liu, Haixia Han, Jie Wang, Dan Hao, Yan Sun

**Affiliations:** 1 Poultry Institute, Shandong Academy of Agricultural Sciences, Jinan 250100, China; lidp1990@126.com (D.L.); lifuwei1224@163.com (F.L.); lwteam@126.com (W.L.); hanhaixia@163.com (H.H.); wangjie4007@126.com (J.W.); haohaodandan111121@gmail.com (D.H.); 2 Shandong Provincial Key Laboratory of Livestock and Poultry Breeding, Jinan 250100, China; 3 Jinan Key Laboratory of Poultry Germplasm Resources Innovation and Healthy Breeding, Jinan 250100, China

**Keywords:** laying hens, cold stress, ammonia exposure, immune response, reproductive function, environmental management

## Abstract

Controlling low ambient temperatures and ammonia levels is critical for effective environmental management in poultry houses during winter, as both represent persistent stressors affecting bird health and productivity. However, evidence regarding their combined long-term effects on the physiological responses of laying hens remains limited. In this study, 576 eighteen-week-old Hy-Line Brown hens were randomly assigned to six treatments (8 replicates with 12 birds per replicate each treatment) and housed in environmentally controlled chambers for 20 weeks: T1 (8 °C, ≤5 ppm ammonia), T2 (8 °C, 20 ppm ammonia), T3 (8 °C, 45 ppm ammonia), T4 (20 °C, ≤5 ppm ammonia; control), T5 (20 °C, 20 ppm ammonia), and T6 (20 °C, 45 ppm ammonia). Plasma samples were collected at 22, 26, 30, 34, and 38 weeks to evaluate physiological stress biomarkers (corticosterone, CORT; total antioxidant capacity, T-AOC), immunoglobulins (IgG, IgM, and IgA), and reproductive hormones (luteinizing hormone, LH; follicle-stimulating hormone, FSH; estradiol, E2). At 38 weeks, hypothalamus, pituitary, and spleen tissues were collected to assess the relative mRNA expression of gonadotropin-releasing hormone (GnRH), FSH, tumor necrosis factor-α (TNF-α), and interleukins (IL-1β, IL-6, and IL-10). Results showed that both cold and ammonia stress reduced antioxidant capacity, disrupted immune homeostasis, and altered reproductive hormone profiles. Cold exposure induced acute immunoendocrine alterations with partial physiological adaptation over time, whereas ammonia exerted progressive and cumulative damage, including elevated immunoglobulins (IgG and IgM) and downregulation of GnRH and FSH expression. Combined exposure significantly upregulated TNF-α and IL-1β expression, suggesting a synergistic inflammatory response. These results highlight complex, parameter-specific interactions between cold and ammonia stressors, emphasizing the need for targeted environmental strategies. Stage-specific interventions—thermal regulation in early laying and ammonia control in later phases—are recommended to safeguard hen health and optimize productivity under winter conditions.

## Simple Summary

Maintaining an optimal indoor environment is essential for laying-hen health and productivity, especially under winter conditions. This study investigated the physiological impacts of prolonged exposure (20 weeks) to low ambient temperature and elevated ammonia concentrations by evaluating stress biomarkers, immune responses, and reproductive function in laying hens. Cold stress elicited acute hormonal and immune responses, followed by partial physiological adaptation, whereas ammonia exposure induced progressive impairments, particularly in immune competence and reproductive performance. Combined exposure further exacerbated inflammatory responses, suggesting a synergistic effect between these stressors. These findings highlight the importance of stage-specific environmental management strategies from a physiological health perspective to optimize hen health and productivity. This research provides practical guidance for improving poultry welfare and advancing sustainable winter farming systems.

## 1. Introduction

Modern commercial poultry houses are enclosed structures. This design allows for precise regulation of environmental parameters, such as temperature and lighting. It also serves as an effective barrier against pathogen introduction by wild birds [1,2]. However, inadequate management or malfunction of environmental control systems can inadvertently expose poultry to adverse conditions, including thermal discomfort, increased ammonia levels, and airborne particulate matter [3,4]. Numerous studies have demonstrated that such environmental stressors negatively impact the productive performance, immune competence, welfare, and overall health of poultry [5,6,7,8,9,10].

Among the various environmental stressors mentioned above, the adverse effects of cold stress on laying hens have been recognized [11,12], although they have been less extensively studied than those of heat stress. Previous research indicated that the thermoneutral zone for optimal metabolic and productive activity in laying hens ranges from 18 °C to 23.9 °C [13]. Exposure to temperatures below 16 °C negatively impacts productive performance by increasing feed intake, reducing egg production and feed efficiency, impairing nutrient digestibility, and decreasing body weight [11,12,14,15]. Furthermore, prolonged exposure to temperatures significantly below this threshold may induce irreversible physiological damage [16].

To mitigate the detrimental effects of cold stress, several nutritional strategies have been proposed. For instance, increasing dietary energy density and supplementing feed with vitamins and minerals can partially alleviate cold-induced stress and support recovery from illness [12,15,17,18,19,20,21]. Nevertheless, these nutritional adjustments are often associated with increased production costs, prompting producers to consider alternative measures. Producers frequently reduce ventilation to conserve heat in poultry houses without heating systems. Although this practice effectively reduces heat loss, it simultaneously increases indoor ammonia concentrations due to inadequate removal of manure emissions [22].

Ammonia is one of the most harmful gases in poultry houses, significantly impairing bird productivity, respiratory health, and welfare [23,24,25,26,27,28]. For instance, chronic exposure to ammonia at concentrations of 106 ppm over seven weeks resulted in reductions of approximately 22.8% in feed intake and 38.7% in daily weight gain [24,29]. Additionally, elevated ammonia levels negatively affect respiratory tract function [30,31], increasing susceptibility to respiratory diseases and secondary infections such as Newcastle disease, tracheitis, and *Mycoplasma gallisepticum* infection [32,33,34]. Moreover, birds exposed to ammonia concentrations of 26 ppm and 52 ppm for 21 days showed significantly reduced hemagglutination inhibition antibody titers against Newcastle disease virus, indicating compromised immune function [35]. These findings underscore the detrimental impact of ammonia on immune response and poultry productivity, although variability exists due to differences in bird age, breed, ammonia concentrations, and duration of exposure.

Maintaining appropriate thermal conditions while ensuring safe ammonia concentrations represents a major challenge in poultry houses, particularly during cold winter months when ventilation is intentionally reduced to conserve heat [13]. Although reduced ventilation effectively mitigates heat loss, it inadvertently elevates ammonia concentrations, potentially exacerbating health and welfare issues in poultry. Despite the recognized importance of these environmental factors, limited research has investigated the combined effects of cold stress and ammonia exposure, particularly with respect to their cumulative impacts on immune response and reproductive physiology in laying hens. Given the extended laying periods—often lasting from 500 to over 700 days [36]—it is essential to systematically assess these combined stressors to enhance long-term poultry welfare and productivity. Therefore, the current study aimed to evaluate both individual and interactive effects of low ambient temperature and elevated ammonia concentrations on immune response and reproductive physiology in laying hens. We hypothesized that the combination of cold stress and high ammonia levels would have a more pronounced negative impact than either stressor alone, potentially leading to immunosuppression and disrupted reproductive function. The findings are expected to provide practical guidance for environmental management strategies aimed at balancing thermal comfort and ammonia control, thereby promoting poultry health, welfare, and productivity under challenging environmental conditions.

## 2. Materials and Methods

### 2.1. Bird Management and Experimental Design

All animal procedures in this study were conducted in accordance with the guidelines approved by the Committee on the Care and Use of Laboratory Animal Resources of the Shandong Academy of Agricultural Sciences (Protocol No. SAAS-2021-026).

A total of 576 Hy-Line Brown laying hens (16 weeks of age) were obtained from a commercial layer farm in China. Prior to the start of the experiment, all birds underwent a 2-week acclimation period in environmentally controlled chambers until 18 weeks of age and were individually labeled by leg tags for subsequent observation.

After acclimation, birds were randomly assigned to six treatment groups, as outlined in Table 1. The experiment employed a 2 × 3 factorial design, with two ambient temperature conditions—thermoneutral (20 ± 2 °C) and cold stress (8 ± 2 °C)—and three ammonia concentrations: ≤5 ppm (low), 20 ppm (moderate), and 45 ppm (high). The ammonia concentrations used in this study (20 ppm and 45 ppm) were selected based on established occupational and agricultural standards. The European Union Directive 2000/39/EC specifies an 8 h time-weighted average (TWA) exposure limit of 14 mg/m^3^ (approximately 20 ppm) and a 15 min short-term exposure limit (STEL) of 36 mg/m^3^ (approximately 50 ppm) under standard conditions (20 °C, 101.3 kPa) [37]. In the United States, the Occupational Safety and Health Administration sets a limit of 50 mg/m^3^ [38], while the National Institute for Occupational Safety and Health recommends a TWA of 25 mg/m^3^ [39]. China’s agricultural industry standard (NY/T 388-1999) sets the maximum allowable ammonia concentration in poultry houses at 15 mg/m^3^ (approximately 20 ppm, 20 °C, 101.3 kPa) [40]. Thus, 20 ppm represents a moderately elevated level within regulatory limits, and 45 ppm simulates high ammonia exposure that may occur in poorly ventilated poultry houses during cold seasons. This design resulted in six treatment combinations (T1–T6). In chambers without supplemental ammonia (T1 and T4), concentrations remained ≤5 ppm due to natural fecal emissions, as monitored by in-room sensors.

Each treatment group consisted of 8 replicates, with 12 birds per replicate. The group exposed to 20 °C and ≤5 ppm ammonia (T4) served as the control (CON). All birds received a standard corn–soybean meal-based diet [41] (Table S1) and had ad libitum access to feed and clean drinking water throughout the experimental period.

In terms of other environmental parameters, indoor RH and carbon dioxide (CO_2_) concentrations were maintained at 40–60% and below 1000 ppm, respectively, throughout the experimental period. For the lighting program, an intensity of 15–25 lx was maintained at bird head level. Starting at 20 weeks of age, birds were initially exposed to 13.5 h of light per day. The duration was then gradually extended each week until it reached 16 h per day at 31 weeks of age. From that point onward, a constant 16 h light schedule was maintained until the end of the experiment.

### 2.2. Environmentally Controlled Chambers

To ensure consistent environmental conditions, the experiment was conducted in six identical, environmentally controlled chambers (each 24.75 m^2^, 4.5 m × 5.5 m). The outer walls of the environmental chambers were made of color-coated steel with a polyurethane core, and the inner walls were constructed from stainless steel. Each chamber was connected to a central control room equipped with a computerized system programmed to regulate environmental parameters, including temperature, relative humidity (RH), atmospheric gas concentrations, and lighting, based on real-time data collected from distributed sensors. Environmental sensors for temperature, humidity, and gas were installed at the center of each chamber to ensure accurate and representative measurements.

A dedicated gas room housed the ammonia delivery system, where compressed anhydrous ammonia was stored in steel cylinders equipped with flow meters to precisely control the release rate. Each chamber was fitted with LED lighting to maintain consistent light intensity and with automated air conditioning units for temperature regulation. Standard feeders and nipple drinkers were provided for all birds, and surveillance cameras were installed in each chamber to allow continuous behavioral monitoring.

### 2.3. Blood Sample Collection and Biochemical Analyses

At 22, 26, 30, 34, and 38 weeks of age, laying hens were fasted for 12 h prior to blood collection. Four birds were randomly selected from each replicate, and blood samples were collected via wing vein puncture. The samples were labeled and centrifuged (20 min, 3000 rpm) to separate plasma, which was subsequently stored at −20 °C until analysis.

Serum concentrations of physiological stress biomarkers (CORT, T-AOC), immunoglobulins (IgG, IgM, and IgA) and reproductive hormones (LH, FSH, and E2) were determined using enzyme-linked immunosorbent assay (ELISA) kits (Table S2, Jianglai Biotechnology Co., Ltd., Shanghai, China). A microplate reader (Rayto RT-6100, Rayto Life and Analytical Sciences Co., Ltd., Shenzhen, China) was used for absorbance measurements. All assays were performed strictly according to the manufacturer’s instructions. To minimize inter-assay variability, all samples were analyzed within a single assay batch.

### 2.4. RNA Isolation, cDNA Synthesis, and Quantitative Real-Time PCR (qRT-PCR) Analysis

At 38 weeks of age, four birds were randomly selected from each replicate (8 replicates per treatment), resulting in 32 birds per treatment group (6 groups, 192 birds in total) for sample collection and analysis. Following a 12 h fasting period, the birds were euthanized by intravenous injection of sodium pentobarbital (100 mg/kg body weight), followed by cervical dislocation to ensure death, and immediately dissected. The hypothalamus, pituitary gland, and spleen were carefully excised, immersed in RNA preservation solution (Cat. No. JL-RRNA50, Jianglai Biotechnology Co., Ltd., Shanghai, China), and snap-frozen in liquid nitrogen to prevent RNA degradation. All samples were then transferred to a −80 °C ultra-low temperature freezer for long-term storage prior to RNA extraction.

Total RNA was extracted using TRIzol reagent (Jianglai Biotechnology Co., Ltd., Shanghai, China) following the manufacturer’s instructions. RNA concentration and purity were assessed using a NanoDrop 2000 spectrophotometer (Thermo Fisher Scientific, Waltham, MA, USA), and only samples meeting quality criteria were used for downstream applications. First-strand complementary DNA (cDNA) was synthesized using a commercial reverse transcription kit (Jianglai Biotechnology Co., Ltd., Shanghai, China) and stored at −20 °C until use.

qRT-PCR was performed using Taq Pro Universal SYBR qPCR Master Mix (Jianglai Biotechnology Co., Ltd., Shanghai, China) on an ABI PRISM 7300 HT sequence detection system (Applied Biosystems, Waltham, MA, USA). The relative mRNA expression levels of GnRH, FSH, TNF-α, IL-1β, IL-6, and IL-10 were quantified. β-actin served as the internal reference gene. Primer sequences were synthesized by Jianglai Biotechnology Co., Ltd., Shanghai, China, and are listed in Table 2. Primer efficiency was determined using a 5-fold serial dilution of pooled cDNA samples. The amplification efficiency for all primers, calculated from the slope of the standard curve, ranged between 95% and 104%. Primer specificity was confirmed by melt curve analysis, which showed a single distinct peak for each gene, and no primer-dimer formation was observed. Relative gene expression levels were calculated using the 2^−ΔΔCt^ method.

### 2.5. Statistical Analysis

A parametric linear mixed model was employed using SPSS software (IBM SPSS Statistics 25.0, Armonk, NY, USA) to evaluate the effects of week of age, ambient temperature, and ammonia concentration, along with their interactions. Replicate was included as a random effect in the model. The model equation was structured as follows:

Y_abcd_ = Δ + WOA_a_ + T_b_ + AC_c_ + WOA × T_ab_ + WOA × AC_ac_ + T × AC_bc_ + R_d_ + δ_abcd_

where Y_abcd_ = observed value for blood parameters or mRNA expression; Δ = overall model constant; WOA_a_ = effect of week of age (a = 1–5); T_b_ = effect of temperature (b = 1–2); AC_c_ = effect of ammonia concentration (c = 1–3); WOA × T_ab_, WOA × AC_ac_, and T × AC_bc_ = interaction terms; R_d_ = effect of replicate (d = 1–8); and δ_abcd_ = residual error term.

When a factor or interaction was found to be non-significant, it was removed from the model. Treatment effects were further evaluated using one-way analysis of variance (ANOVA), and mean differences among treatment groups were compared using Duncan’s multiple range test. Results are presented as mean ± SEM, and differences were considered statistically significant at *p* < 0.05.

## 3. Results

### 3.1. CORT and T-AOC

As shown in Table 3, exposure to a low ambient temperature (8 °C) led to a numerical increase (1.78%) in plasma CORT levels compared to the thermoneutral temperature (20 °C). In contrast, cold stress significantly reduced plasma T-AOC by 9.22% (*p* < 0.05). Exposure to 45 ppm ammonia significantly elevated CORT levels by approximately 6.7% compared to both lower concentrations (*p* < 0.05). Moreover, T-AOC levels were significantly decreased in hens exposed to 20 ppm ammonia compared to those exposed to ≤5 ppm (*p* < 0.05), with a further reduction observed at 45 ppm (*p* < 0.05).

**Figure 1:**
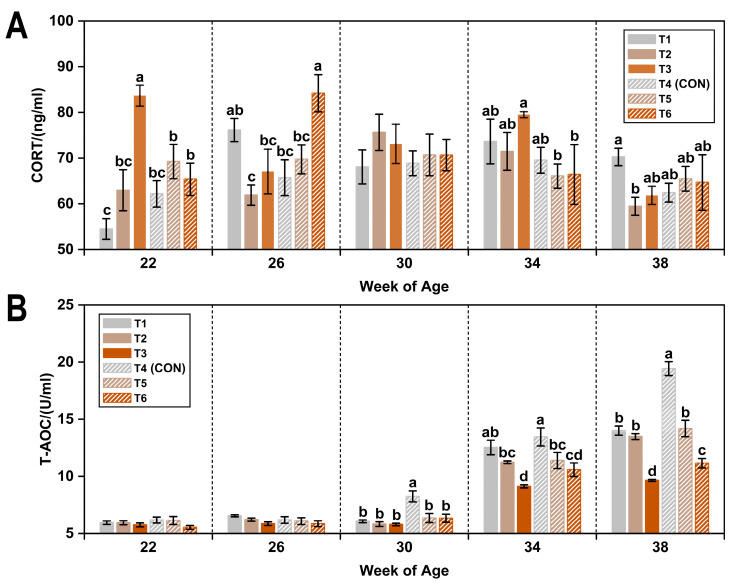
Plasma CORT (**A**) and T-AOC (**B**) levels in laying hens exposed to different ambient temperatures and ammonia concentrations from 22 to 38 weeks of age. Treatments T1–T6 represent six experimental groups: T1 (8 °C, ≤5 ppm ammonia), T2 (8 °C, 20 ppm ammonia), T3 (8 °C, 45 ppm ammonia), T4 (20 °C, ≤5 ppm ammonia; CON), T5 (20 °C, 20 ppm ammonia), and T6 (20 °C, 45 ppm ammonia). Values with different superscript letters at the same week of age differ significantly (*p* < 0.05); values sharing the same letter are not significantly different. Data are presented as mean ± SEM.

Figure 1 illustrates the temporal variations in plasma CORT and T-AOC under varying temperature and ammonia conditions. Plasma CORT concentrations increased with age, peaking at 30 weeks (71.19 ± 1.54 ng/mL), and subsequently declined. CORT levels from 26 to 34 weeks were significantly higher than those at 22 and 38 weeks (*p* < 0.05). In contrast, plasma T-AOC levels exhibited a continuous age-related increase, with a significant increase at 34 weeks and a peak at 38 weeks (*p* < 0.05).

### 3.2. Immunoglobulins

Low temperature and ammonia concentration exerted distinct effects on plasma immunoglobulin levels in laying hens (Table 4). Compared with the thermoneutral condition (20 °C), exposure to a low temperature (8 °C) significantly increased plasma IgG and IgM levels by 23.7% and 16.4%, respectively (*p* < 0.05), while significantly decreasing IgA by 9.7% (*p* < 0.05).

Regarding the effects of ammonia, IgM concentrations were significantly higher in hens exposed to 20 ppm and 45 ppm ammonia compared with those receiving ≤5 ppm (*p* < 0.05). In contrast to the response under low temperature, increasing ammonia levels resulted in elevated plasma IgA concentrations, although a significant increase was only observed at 45 ppm relative to both lower concentrations (*p* < 0.05).

**Figure 2:**
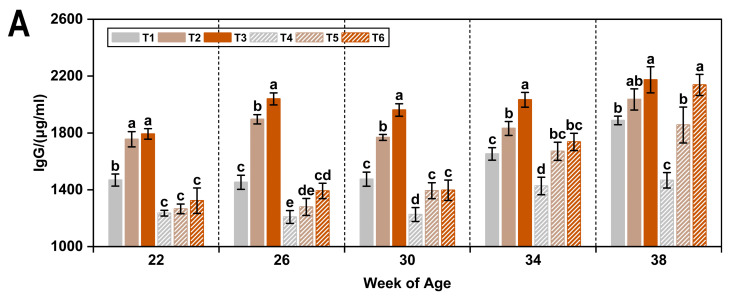

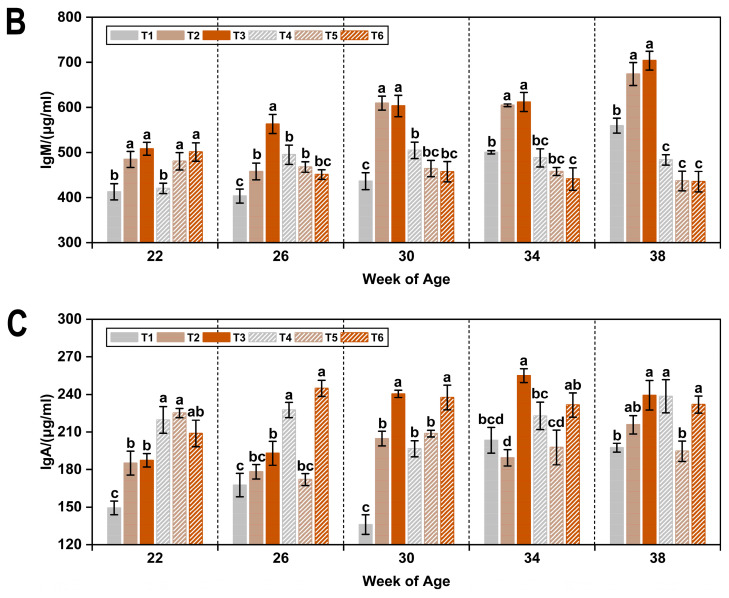
Plasma IgG (**A**), IgM (**B**), and IgA (**C**) levels in laying hens exposed to different ambient temperatures and ammonia concentrations from 22 to 38 weeks of age. Treatments T1–T6 represent six experimental groups: T1 (8 °C, ≤5 ppm ammonia), T2 (8 °C, 20 ppm ammonia), T3 (8 °C, 45 ppm ammonia), T4 (20 °C, ≤5 ppm ammonia; CON), T5 (20 °C, 20 ppm ammonia), and T6 (20 °C, 45 ppm ammonia). Values with different superscript letters at the same week of age differ significantly (*p* < 0.05); values sharing the same letter are not significantly different. Data are presented as mean ± SEM.

As shown in Figure 2, plasma IgG levels in hens exposed to a low temperature and low ammonia concentration (T1) were significantly higher than those under thermoneutral conditions (T4) at 22 weeks of age (*p* < 0.05). Under thermoneutral conditions (20 °C), increasing ammonia concentrations (20 ppm and 45 ppm) significantly elevated plasma IgG levels from 26 weeks onward, with the greatest difference observed at 38 weeks (*p* < 0.05). Under low-temperature conditions (8 °C), IgM levels increased significantly in response to elevated ammonia concentrations (*p* < 0.05).

### 3.3. Reproductive Hormones

According to the results presented in Table 5, exposure to low ambient temperature significantly reduced plasma concentrations of LH, FSH, and E2 by 9.6%, 7.0%, and 4.4%, respectively (*p* < 0.05).

These reproductive hormone levels also declined with increasing ammonia concentrations. Specifically, compared with the clean air group (≤5 ppm ammonia), LH concentrations decreased significantly by 7.7% at 20 ppm and by 9.7% at 45 ppm (*p* < 0.05). Similar decreasing trends were observed for both FSH and E2.

**Figure 3:**
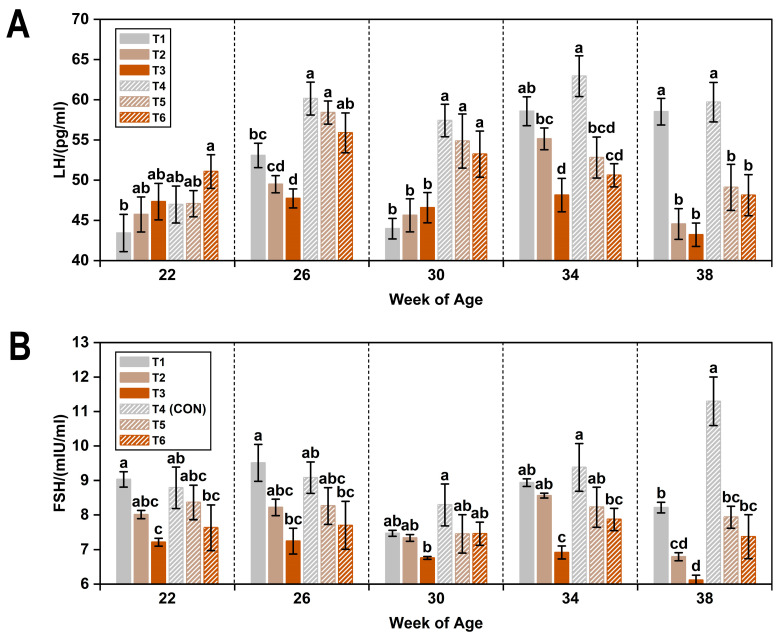

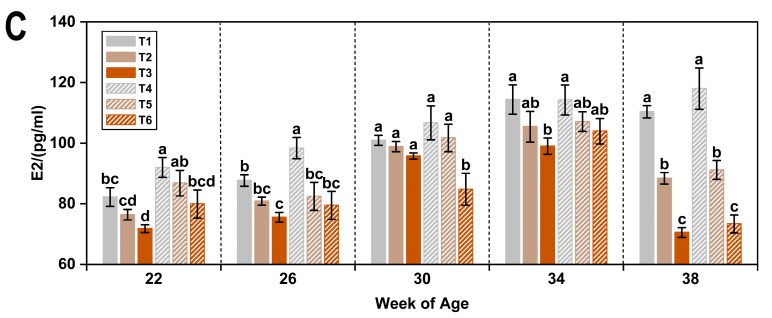
Plasma LH (**A**), FSH (**B**), and E2 (**C**) levels in laying hens exposed to different ambient temperatures and ammonia concentrations from 22 to 38 weeks of age. Treatments T1–T6 represent six experimental groups: T1 (8 °C, ≤5 ppm ammonia), T2 (8 °C, 20 ppm ammonia), T3 (8 °C, 45 ppm ammonia), T4 (20 °C, ≤5 ppm ammonia; CON), T5 (20 °C, 20 ppm ammonia), and T6 (20 °C, 45 ppm ammonia). Values with different superscript letters at the same week of age differ significantly (*p* < 0.05); values sharing the same letter are not significantly different. Data are presented as mean ± SEM.

Changes in plasma reproductive hormone concentrations across the six treatments over time are illustrated in Figure 3. At 34 and 38 weeks of age, plasma LH levels were significantly lower in hens exposed to elevated ammonia concentrations (20 ppm and 45 ppm) compared to those in the low ammonia groups (T1 and CON) (*p* < 0.05). At 22 weeks of age, plasma FSH levels subjected to low temperature combined with 45 ppm ammonia were also significantly reduced relative to T1 and CON groups (*p* < 0.05). Plasma E_2_ concentrations increased gradually across all treatments until 34 weeks of age. However, as observed for LH, exposure to 20 ppm and 45 ppm ammonia significantly suppressed E2 levels at 38 weeks compared to the low ammonia treatments (*p* < 0.05).

### 3.4. Gene Expression

As shown in Table 6, both GnRH and FSH expression levels significantly decreased under low temperature and 45 mg/kg ammonia (*p* < 0.05).

**Figure 4:**
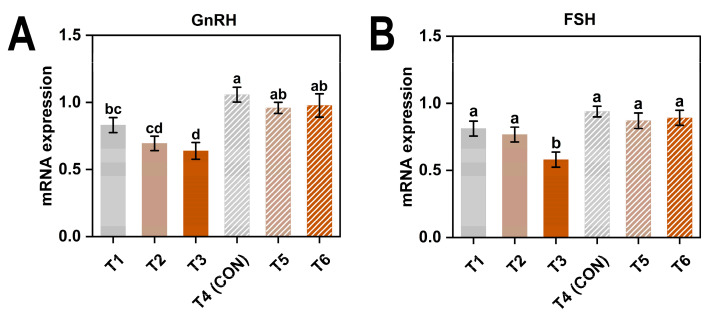
Gene expression levels of GnRH (**A**) and FSH (**B**) in laying hens exposed to different ambient temperatures and ammonia concentrations at 38 weeks of age. Treatments T1–T6 represent six experimental groups: T1 (8 °C, ≤5 ppm ammonia), T2 (8 °C, 20 ppm ammonia), T3 (8 °C, 45 ppm ammonia), T4 (20 °C, ≤5 ppm ammonia; CON), T5 (20 °C, 20 ppm ammonia), and T6 (20 °C, 45 ppm ammonia). Values with different superscript letters differ significantly (*p* < 0.05); values sharing the same letter are not significantly different. Data are presented as mean ± SEM.

As shown in Figure 4, the expression levels of GnRH and FSH were generally higher in hens exposed to thermoneutral conditions compared to those under cold stress. Birds in the T3 (8 °C, 45 ppm) exhibited significantly lower expression levels of both genes compared with any of the thermoneutral groups (*p* < 0.05), suggesting a pronounced inhibitory effect of the combined cold and high ammonia exposure on reproductive gene expression.

Exposure to low temperature and excessive ammonia significantly increased the relative expression levels of TNF-α and IL-1β (*p* < 0.05) (Table 7). Additionally, there was a significant interaction between temperature and ammonia concentration affecting the expression of both genes (*p* < 0.05).

**Figure 5:**
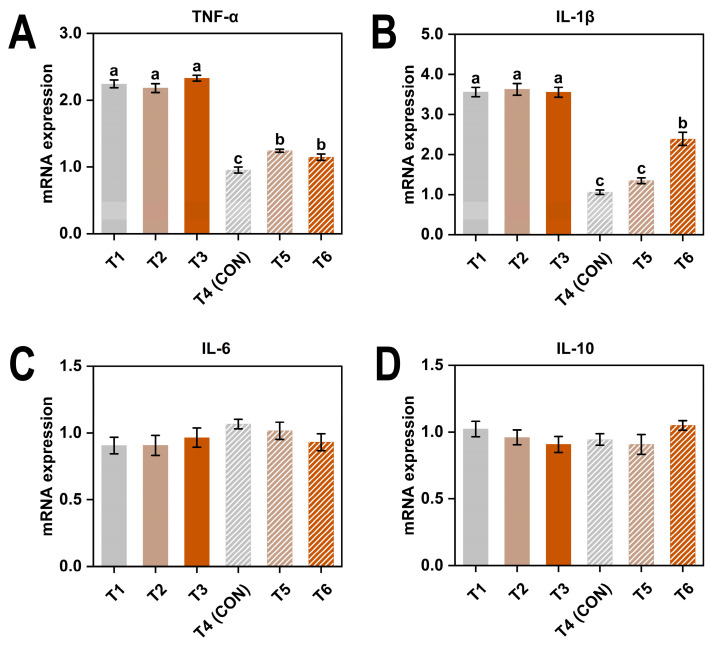
Gene expression levels of TNF-α (**A**), IL-1β (**B**), IL-6 (**C**), and IL-10 (**D**) in laying hens exposed to different ambient temperatures and ammonia concentrations at 38 weeks of age. Treatments T1–T6 represent six experimental groups: T1 (8 °C, ≤5 ppm ammonia), T2 (8 °C, 20 ppm ammonia), T3 (8 °C, 45 ppm ammonia), T4 (20 °C, ≤5 ppm ammonia; CON), T5 (20 °C, 20 ppm ammonia), and T6 (20 °C, 45 ppm ammonia). Values with different superscript letters differ significantly (*p* < 0.05); values sharing the same letter are not significantly different. Data are presented as mean ± SEM.

According to Figure 5A,B, under thermoneutral conditions, higher ammonia concentrations led to a significant upregulation of both genes (*p* < 0.05).

## 4. Discussion

Environmental stress is a persistent challenge in modern poultry farming, especially under enclosed and high-density commercial conditions [1]. Such environments frequently lead to thermal discomfort and compromised air quality [13,42]. Historically, research has primarily focused on the isolated effects of individual environmental stressors—such as temperature fluctuations or elevated ammonia concentrations—on poultry physiology and productive performance [9,22,35,43]. However, despite the frequent common co-occurrence of cold temperatures and high ammonia levels during winter housing, few studies have systematically investigated their interactive effects. In recent years, growing attention to animal welfare and sustainable production practices has expanded research interests to include the influence of environmental stress on immune and endocrine regulatory mechanisms [44,45]. Addressing this gap, the present study aims to evaluate how prolonged exposure to low ambient temperature and different levels of ammonia affects the physiological homeostasis of laying hens.

### 4.1. Physiological Stress Biomarkers

Corticosterone is widely recognized as a biomarker for assessing stress in poultry, as it reflects activation of the hypothalamic–pituitary–adrenal (HPA) axis and plays a dual role in regulating immune function and maintaining metabolic homeostasis [46,47]. Previous studies have demonstrated a negative correlation between elevated CORT levels and physiological health. For instance, Calefi et al. [48] reported that increased CORT concentrations were associated with immune dysfunction, which mirrors the immunosuppressive trends observed in the present study. Additionally, exogenous CORT administration has been shown to inhibit normal physiological processes in poultry, ultimately reducing productive performance [47]. Similar response patterns have also been documented in studies involving heat-stressed birds [49,50]. However, in the current study, hens exposed to 8 °C and 20 ppm ammonia did not exhibit significant changes in CORT concentrations, indicating that moderate, long-term environmental stress may be insufficient to elicit a consistent endocrine response. Consequently, the irregular fluctuations in CORT levels observed beyond 20 weeks highlight its limitations as a reliable physiological biomarker for chronic environmental stress, underscoring the necessity for standardized approaches in stress assessment [51].

In contrast, T-AOC—an integrative indicator of both enzymatic and non-enzymatic antioxidant defense mechanisms—exhibited more stable and interpretable trends [42,46,47,48,49,50,52,53]. Previous studies have demonstrated that oxidative stress is a key component of the physiological response in birds subjected to extreme temperatures and other environmental stressors [54,55]. It has been reported that individuals possessing higher antioxidant capacities are better equipped to mitigate oxidative damage caused by environmental challenges [56,57]. In the current study, exposure to low ambient temperature and ammonia concentrations exceeding 20 ppm significantly reduced plasma T-AOC levels, indicative of oxidative stress. These findings are consistent with previous research that identified redox imbalance as a hallmark of chronic environmental exposure [55,58,59]. T-AOC levels increased progressively with age across all experimental groups. This trend likely reflects the physiological maturation of antioxidant systems. However, the increase was markedly attenuated in stress-exposed hens. This suggests that prolonged exposure to environmental stress may impair the development of oxidative defense mechanisms. The present results agree with findings from comparable studies in ducks, in which acute exposure to extreme temperature stress over 28 days also resulted in significantly lower T-AOC levels compared with birds maintained at 23 °C [60].

### 4.2. Immune Responses to Cold and Ammonia Stress

Immunoglobulins—IgG, IgM, and IgA—are essential components of the humoral immune system, serving as crucial biomarkers of immune function [61,62,63]. Previous studies have reported that environmental stressors can elevate IgG levels in poultry [43,64,65]. In the present study, cold stress was found to significantly increase plasma IgG and IgM concentrations while concurrently decreasing IgA levels, suggesting a shift from mucosal toward systemic immune defense. This observation is partially supported by the findings of Hangalapura et al. [66], who reported no significant alterations in antibody production under short-term cold exposure, indicating that both the duration of environmental stress and the genetic background of the birds significantly influence immunological outcomes.

Ammonia exposure elicited complex immunomodulatory effects. For example, chronic exposure (45 weeks) to ammonia concentrations of 30 ppm reportedly decreased plasma IgM levels in hens [43]. Similarly, in the current study, prolonged exposure to ammonia at 45 ppm significantly suppressed IgM levels while upregulating IgA. This upregulation of IgA may be attributed to mucosal irritation caused by prolonged ammonia exposure, particularly in the respiratory tract. Such irritation can activate local immune cells and promote secretory IgA production at mucosal surfaces, serving as a primary defense mechanism against inhaled pathogens and irritants. High concentrations of ammonia have been shown to cause damage to the respiratory mucosa, including ciliary loss and epithelial necrosis, leading to increased susceptibility to respiratory infections [67]. The mucosal immune system responds to such irritants by enhancing IgA production, which plays a crucial role in neutralizing pathogens and maintaining mucosal homeostasis [68]. Therefore, the observed increase in IgA levels in ammonia-exposed hens likely reflects a compensatory mucosal immune response to counteract the detrimental effects of ammonia-induced respiratory tract irritation. These findings are consistent with Duan et al. [69], who associated ammonia inhalation with immune dysfunction and inflammatory responses. Notably, there was no significant difference in IgG levels among hens from treatments T4 to T6 at 22 weeks of age, suggesting that elevated ammonia concentrations under thermoneutral conditions may not substantially influence immune parameters during the early stage of the laying cycle.

Cytokines play a critical role in regulating B cell activity and immunoglobulin production. They influence various stages of the humoral immune response, including B cell proliferation, differentiation into plasma cells, and antibody class switching. Through these mechanisms, cytokines help shape the quantity and type of immunoglobulins produced under different physiological and environmental conditions [70]. Under environmental stress, alterations in cytokine profiles may affect immunoglobulin dynamics, contributing to shifts in systemic and mucosal immune responses. From an immunological perspective, the expression of pro-inflammatory cytokines TNF-α and IL-1β was significantly increased under both low temperature and high ammonia exposure, with a notable interaction effect observed. This suggests that these two stressors may exacerbate inflammatory responses when present simultaneously, potentially leading to chronic immune activation and tissue damage [43]. This cytokine response pattern appears to differ from that typically observed under heat stress, which is often characterized by a more pronounced upregulation of pro-inflammatory cytokines alongside suppression of anti-inflammatory cytokines such as IL-10 [71]. In contrast, our findings showed relatively stable expression levels of IL-6 and IL-10 across treatments, indicating a more moderate or selectively regulated immune response to cold and ammonia exposure. This differential expression profile highlights the distinct immunological signatures elicited by different environmental stressors and aligns with previous reports suggesting that stress may trigger milder but sustained inflammatory responses, possibly through unique signaling pathways [72,73]. These findings suggest that the immune system exhibits stressor-specific reactivity and that cytokine expression patterns should be interpreted in the context of the type, duration, and intensity of environmental challenges.

Temporal analysis further revealed that ammonia-induced immunosuppression became increasingly pronounced during later laying stages, whereas the immunological impact of cold stress was more immediate but transient. This differential temporal pattern may result from the progressive accumulation of ammonia, contributing to cumulative physiological burden. Collectively, these findings indicate that the variation trends in immunoglobulin levels are likely governed by the type, intensity, and duration of environmental stress [13,42]. The present study highlights that, over time, ammonia exposure exerts more pronounced effects on the humoral immune system of laying hens compared to cold stress.

### 4.3. Reproductive Hormonal Regulation

The reproductive performance of laying hens is tightly regulated by the hypothalamic–pituitary–gonadal (HPG) axis through hormones such as LH, FSH, and E2 [74,75]. Our findings showed that both low temperature and ammonia exposure significantly reduced the circulating levels of these hormones. Importantly, no interaction effects between temperature and ammonia were observed, suggesting that each stressor independently disrupts endocrine regulation. Specifically, cold stress primarily suppresses LH and FSH during early laying stages, potentially delaying sexual maturation or impairing peak productivity. Novero et al. [76] previously demonstrated that exposure to a high temperature of 35 °C reduced plasma levels of LH and progesterone, providing a valuable reference for the present study. This suggests that a low temperature of 8 °C may also act as a thermal stressor, similarly impairing the normal secretion of LH by the pituitary gland.

In contrast, ammonia exposure exerted more pronounced effects during later laying stages, possibly due to its cumulative impact on pituitary and ovarian function [77]. In general, ammonia had no significant impact on LH levels at the same temperature before 30 weeks of age, whereas low temperature exerted a more prominent inhibitory effect during this early phase. As the laying period progressed, the influence of ammonia on LH became increasingly pronounced, and a similar trend was observed for FSH. Reduced LH and FSH likely impaired E2 synthesis, consequently compromising follicular development and reproductive performance. Correspondingly, E2 levels of birds exposed to low temperature and ammonia concentrations exceeding 20 ppm were significantly lower than those in CON, likely as a result of suppressed gonadotropin secretion [78,79,80]. Similar hormonal disruptions have been documented in studies examining heat stress and air pollution effects [42,78]. Additionally, Wolfenson et al. [81] reported that heat stress could reduce ovarian blood flow, further impairing hormone synthesis and oocyte quality. This mechanism may also help explain the markedly lower E2 levels observed in hens exposed to low temperature and high ammonia concentrations in the present study.

In addition to the direct impact of environmental stressors on gonadotropins, stress-induced hormonal changes may also be mediated through CORT, the primary glucocorticoid in birds [82]. CORT plays a pivotal role in the physiological response to stress, and elevated levels have been shown to suppress reproductive function by interfering with the HPG axis [83]. Specifically, increased plasma CORT concentrations can downregulate the secretion of key reproductive hormones, leading to impaired follicular development and reduced egg production [84]. Furthermore, chronic elevation of CORT under stress conditions may alter energy allocation, prioritizing survival mechanisms over reproductive processes [85]. These findings underscore the intricate interplay between stress and reproductive endocrinology in poultry and provide a potential mechanistic explanation for the observed hormonal disruptions in our study.

### 4.4. Reproductive-Related Gene Expression Patterns

In the present study, both low ambient temperature (8 °C) and high ammonia exposure (45 ppm) significantly downregulated the expression levels of GnRH and FSH in laying hens, indicating that these environmental stressors independently impair neuroendocrine function at the molecular level. The lack of interaction between temperature and ammonia suggests additive, rather than synergistic, suppression of the reproductive axis. These findings are consistent with previous work indicating that cold and air pollutants inhibit hypothalamic and pituitary gene expression, thereby reducing gonadotropin secretion [86,87]. Considering the changes in circulating CORT observed in this study, it is plausible that the suppression of reproductive hormones is at least partially mediated by stress-induced glucocorticoid signaling. These results align with mammalian research showing that glucocorticoids can affect gonadal function at multiple regulatory levels [88]. Specifically, glucocorticoids can suppress the synthesis and release of GnRH from the hypothalamus by disrupting its pulsatile secretion pattern [89] and can also reduce circulating levels of LH and FSH by inhibiting pituitary responsiveness to GnRH [90]. This supports the notion that environmental stress may interfere with neuroendocrine signaling through both hypothalamic and pituitary pathways.

Collectively, the current findings demonstrate that combined cold and ammonia stress not only disrupts endocrine pathways governing reproduction but also alters immune gene expression profiles in a complex manner. Such transcriptional dysregulation likely underlies the physiological impairments observed in laying hens exposed to long-term environmental challenges.

Future studies should explore extended exposure durations and systematically investigate interactions among multiple environmental factors—including temperature, ammonia, RH, and other relevant parameters—to comprehensively understand their physiological impacts throughout the entire laying cycle. Collectively, these findings offer a scientific foundation for optimizing environmental management to ensure poultry health, welfare, and sustained productivity from a physiological health perspective.

## 5. Conclusions

This study demonstrated that both cold stress and elevated ammonia exert statistically significant effects on key physiological indicators in laying hens, including corticosterone levels, antioxidant capacity, immune responses, and reproductive hormone secretion. Notably, hens showed partial physiological adaptation to prolonged cold exposure, whereas the adverse effects of ammonia accumulated progressively, indicating cumulative physiological damage over time.

These findings provide critical insights for environmental management strategies in poultry production, particularly during winter conditions. They highlight the necessity of stage-specific regulation: thermal control should be prioritized during the early laying phase to prevent cold-induced suppression, while stringent ammonia management (below 20 ppm) should be emphasized in the later stages to limit chronic physiological impairments.

## Figures and Tables

**Table 1:** Design of experimental treatments.

Item ^1^	T1	T2	T3	T4 (CON)	T5	T6
T (°C)	8	8	8	20	20	20
AC (ppm)	≤5	20	45	≤5	20	45

Note: ^1^ T: temperature; AC: ammonia concentration.

**Table 2:** qRT-PCR primer sequences.

Target Gene	Primer	Primer Sequences (5′-3′)	Product Length (bp)
β-actin	Forward	CACGATCATGTTTGAGACCTT	100
	Reverse	CATCACAATACCAGTGGTACG	
FSH	Forward	TTATGTAGCCCACTGAGGAAAGCCA	257
	Reverse	TAGCTGAGCCGTTCTACACTTTGGA	
GnRH	Forward	AATCTGCTTGGCTCAACACTG	220
	Reverse	AATCTCCTTTCTTCTGGCTTCT	
TNF-α	Forward	ATGAACCCTCCGCAGTACTC	200
	Reverse	AAGAGGCCACCACACGACA	
IL-1β	Forward	GGGCTACAAGCTCTACATGT	118
	Reverse	GTAGAAGATGAAGCGGGTCA	
IL-6	Forward	AGAAATCCCTCCTCGCCAAT	121
	Reverse	AAATAGCGAACGGCCCTCA	
IL-10	Forward	CGCTGTCACCGCTTCTTCA	88
	Reverse	TCCCGTTCTCATCCATCTTCTC	

**Table 3:** Effect of week of age, temperature, and ammonia concentration on plasma CORT and T-AOC levels in laying hens.

Item ^1^	CORT (ng/mL)	T-AOC (U/mL)
WOA		
22	66.33 ± 1.68 ^b^	5.92 ± 0.09 ^c^
26	70.80 ± 1.66 ^a^	6.13 ± 0.09 ^c^
30	71.19 ± 1.54 ^a^	6.44 ± 0.16 ^c^
34	71.11 ± 1.69 ^a^	11.39 ± 0.28 ^b^
38	64.03 ± 1.31 ^b^	13.65 ± 0.41 ^a^
T (°C)		
8	69.30 ± 1.01	8.27 ± 0.23 ^b^
20	68.09 ± 1.03	9.11 ± 0.32 ^a^
AC (ppm)		
≤5	67.14 ± 1.09 ^b^	9.86 ± 0.43 ^a^
20	67.27 ± 1.14 ^b^	8.69 ± 0.32 ^b^
45	71.66 ± 1.45 ^a^	7.57 ± 0.22 ^c^
*p*-value ^2^		
WOA	<0.05	<0.05
T	NS	<0.05
AC	<0.05	<0.05
WOA × T	NS	<0.05
WOA × AC	<0.05	<0.05
T × AC	NS	<0.05

Note: Values are presented as mean ± SEM. Values within a column bearing different superscript letters differ significantly (*p* < 0.05); values sharing the same letter are not significantly different. ^1^ WOA: week of age; T: temperature; AC: ammonia concentration. ^2^ NS: not significant (*p* > 0.05).

**Table 4:** Effects of week of age, temperature, and ammonia concentration on plasma immunoglobulin levels in laying hens.

Item ^1^	IgG (μg/mL)	IgM (μg/mL)	IgA (μg/mL)
WOA			
22	1473.51 ± 33.46 ^c^	468.02 ± 8.09 ^c^	195.89 ± 4.40 ^b^
26	1544.36 ± 41.55 ^c^	473.12 ± 8.87 ^c^	197.19 ± 4.47 ^b^
30	1536.65 ± 35.79 ^c^	512.62 ± 11.31 ^b^	203.99 ± 4.83 ^b^
34	1725.28 ± 30.95 ^b^	517.44 ± 10.21 ^b^	216.61 ± 4.71 ^a^
38	1925.78 ± 42.42 ^a^	548.86 ± 15.07 ^a^	219.58 ± 4.18 ^a^
T (°C)			
8	1814.70 ± 20.48 ^a^	542.29 ± 8.08 ^a^	196.12 ± 3.01 ^b^
20	1467.53 ± 25.32 ^b^	465.74 ± 4.91 ^b^	217.18 ± 2.64 ^a^
AC (ppm)			
≤5	1449.88 ± 22.87 ^c^	470.48 ± 6.61 ^b^	195.88 ± 4.03 ^b^
20	1675.12 ± 30.34 ^b^	513.86 ± 8.90 ^a^	197.12 ± 2.70 ^b^
45	1798.35 ± 34.41 ^a^	527.69 ± 10.05 ^a^	226.96 ± 3.21 ^a^
*p*-value ^2^			
WOA	<0.05	<0.05	<0.05
T	<0.05	<0.05	<0.05
AC	<0.05	<0.05	<0.05
WOA × T	<0.05	<0.05	<0.05
WOA × AC	NS	NS	<0.05
T × AC	NS	<0.05	<0.05

Note: Values are presented as mean ± SEM. Values within a column bearing different superscript letters differ significantly (*p* < 0.05); values sharing the same letter are not significantly different. ^1^ WOA: week of age; T: temperature; AC: ammonia concentration. ^2^ NS: not significant (*p* > 0.05).

**Table 5:** Effect of week of age, temperature, and ammonia concentration on plasma reproductive hormone levels in laying hens.

Item ^1^	LH (pg/mL)	FSH (mIU/mL)	E2 (pg/mL)
WOA			
22	46.94 ± 0.89 ^c^	8.17 ± 0.19 ^a^	81.50 ± 1.51 ^d^
26	54.12 ± 0.85 ^a^	8.33 ± 0.21 ^a^	84.04 ± 1.55 ^d^
30	50.28 ± 1.10 ^b^	7.46 ± 0.15 ^b^	98.11 ± 1.70 ^b^
34	54.70 ± 0.99 ^a^	8.31 ± 0.18 ^a^	107.33 ± 1.80 ^a^
38	50.54 ± 1.15 ^b^	7.95 ± 0.26 ^ab^	91.94 ± 2.50 ^c^
T (°C)			
8	48.74 ± 0.58 ^b^	7.76 ± 0.09 ^b^	90.52 ± 1.20 ^b^
20	53.89 ± 0.69 ^a^	8.34 ± 0.16 ^a^	94.64 ± 1.49 ^a^
AC (ppm)			
≤5	54.47 ± 0.88 ^a^	9.00 ± 0.17 ^a^	102.47 ± 1.64 ^a^
20	50.28 ± 0.77 ^b^	7.92 ± 0.12 ^b^	91.89 ± 1.40 ^b^
45	49.19 ± 0.71 ^b^	7.23 ± 0.13 ^c^	83.39 ± 1.46 ^c^
*p*-value ^2^			
WOA	<0.05	<0.05	<0.05
T	<0.05	<0.05	<0.05
AC	<0.05	<0.05	<0.05
WOA × T	<0.05	<0.05	NS
WOA × AC	<0.05	<0.05	<0.05
T × AC	NS	NS	NS

Note: Values are presented as mean ± SEM. Values within a column bearing different superscript letters differ significantly (*p* < 0.05); values sharing the same letter are not significantly different. ^1^ WOA: week of age; T: temperature; AC: ammonia concentration. ^2^ NS: not significant (*p* > 0.05).

**Table 6:** Effect of temperature and ammonia concentration on mRNA expression levels of GnRH and FSH in laying hens.

Item ^1^	GnRH	FSH
T (°C)		
8	0.72 ± 0.16 ^b^	0.73 ± 0.16 ^b^
20	0.99 ± 0.15 ^a^	0.90 ± 0.12 ^a^
AC (ppm)		
≤5	0.94 ± 0.18 ^a^	0.88 ± 0.13 ^a^
20	0.83 ± 0.18 ^ab^	0.82 ± 0.14 ^ab^
45	0.81 ± 0.25 ^b^	0.74 ± 0.21 ^b^
*p*-value ^2^		
T	<0.05	<0.05
AC	<0.05	<0.05
T × AC	NS	NS

Note: Values are presented as mean ± SEM. Values within a column bearing different superscript letters differ significantly (*p* < 0.05); values sharing the same letter are not significantly different. ^1^ T: temperature; AC: ammonia concentration. ^2^ NS: not significant (*p* > 0.05).

**Table 7:** Effect of temperature and ammonia concentration on mRNA expression levels of inflammatory cytokines in laying hens.

Item ^1^	TNF-α	IL-1β	IL-6	IL-10
T (°C)				
8	2.25 ± 0.15 ^a^	3.58 ± 0.30 ^a^	0.93 ± 0.16	0.96 ± 0.14
20	1.11 ± 0.15 ^b^	1.60 ± 0.64 ^b^	1.00 ± 0.14	0.97 ± 0.14
AC (ppm)				
≤5	1.60 ± 0.69 ^b^	2.31 ± 1.32 ^b^	0.99 ± 0.15	0.98 ± 0.13
20	1.71 ± 0.50 ^a^	2.50 ± 1.22 ^b^	0.96 ± 0.17	0.93 ± 0.16
45	1.74 ± 0.63 ^a^	2.97 ± 0.70 ^a^	0.95 ± 0.16	0.98 ± 0.14
*p*-value ^2^				
T	<0.05	<0.05	NS	NS
AC	<0.05	<0.05	NS	NS
T × AC	<0.05	<0.05	NS	NS

Note: Values are presented as mean ± SEM. Values within a column bearing different superscript letters differ significantly (*p* < 0.05); values sharing the same letter are not significantly different. ^1^ T: temperature; AC: ammonia concentration. ^2^ NS: not significant (*p* > 0.05).

## Data Availability

The original contributions presented in this study are included in the article/Supplementary Materials. Further inquiries can be directed to the corresponding authors.

## Supplementary Materials

The following supporting information can be downloaded at https://www.mdpi.com/article/10.3390/ani15121769/s1, Table S1: Ingredients and nutrient levels of corn–soybean meal-based diet (air-dry basis); Table S2: Information on ELISA kits used in this study.

## Abbreviations

The following abbreviations are used in this manuscript:

CORT	Corticosterone
T-AOC	Total Antioxidant Capacity
Ig	Immunoglobulin
LH	Luteinizing Hormone
FSH	Follicle-stimulating Hormone
E2	Estradiol
GnRH	Gonadotropin-releasing Hormone
TNF-α	Tumor Necrosis Factor-α
IL	Interleukin
TWA	Time-weighted Average
STEL	Short-term Exposure Limit
RH	Relative Humidity
CON	Control
ELISA	Enzyme-linked Immunosorbent Assay
WOA	Week of Age
AC	Ammonia Concentration
NS	Not Significant
HPA	Hypothalamic–Pituitary–Adrenal
HPG	Hypothalamic–Pituitary–Gonadal
