# Supplementary material for: Physiological Responses of Laying Hens to Chronic Cold Stress and Ammonia Exposure: Implications for Environmental Management and Poultry Welfare
Source: Animals (Basel). 2025 Jun 16;15(12):1769. doi: 10.3390/ani15121769 (PMC12189347; doi:10.3390/ani15121769)
Supplement: Supplementary file 1 [file animals-15-01769-s001.zip › animals-3667949-supplementary.pdf]

Table S1. Ingredients and nutrient levels of corn–soybean meal-based diet (air-dry basis).

Item	18-20 WOA[1]	21-38 WOA
Ingredient (%)		
Corn	68.00	65.50
Soybean meal	24.00	23.50
Limestone	—	3.00
Calcareous granule	6.00	6.00
Zeolite powder	0.23	0.23
Dicalcium phosphate	0.105	0.105
Monocalcium phosphate	0.595	0.595
Sodium chloride	0.25	0.25
50% Choline chloride	0.12	0.12
DL-methionine	0.20	0.20
Premix of vitamins and trace minerals[2]	0.50	0.50
Nutrient content (calculated)		
AME_n (kcal/kg)	2800	2700
Crude protein (%)	16.50	16.00
Total amino acid (%)		
Lysine	0.84	0.82
Methionine	0.47	0.47
Methionine + Cystine	0.74	0.73
L-tryptophan	0.19	0.19
Threonine	0.62	0.61
Calcium (%)	2.60	3.80
Total phosphorus (%)	0.50	0.50
Nonphytate phosphorus (%)	0.27	0.27
Nutrient content (measured)		
DM (%)	91.61	92.08
Gross energy (kcal/kg)	3944	3892
Crude protein (%)	16.59	16.04
Total amino acid (%)		
Lysine	0.81	0.79
Methionine	0.48	0.45
Methionine + Cystine	0.75	0.70
L-tryptophan	0.20	0.18
Threonine	0.59	0.59
Calcium (%)	2.68	3.78
Total phosphorus (%)	0.51	0.54

Note: [1]WOA: weeks of age; [2]Premix contained (diet in kg): selenium, 0.3 mg; vitamin A, 7715 IU; copper, 8 mg; vitamin D_3, 2755 IU; zinc, 80 mg; vitamin E, 8.8 IU; biotin, 0.20 mg; cobalamin, 20 μg; pyridoxine, 3.25 mg; riboflavin, 2.21 mg; menadione, 2.2 mg; nicotinic acid, 19.8 mg; folic acid, 0.28 mg; thiamine, 0.65 mg; pantothenic acid, 3.51 mg; manganese, 65 mg; iron, 60 mg; and iodine, 1.0 mg.

Table S2. Information on ELISA kits used in this study.

Target Analyte	Kit Name	Catalog Number	Detection Range	Sensitivity
CORT	Chicken Corticosterone (CORT) ELISA Kit	JLGR-E60114	5-160 ng/mL	1.0 ng/mL
T-AOC	Chicken Total Antioxidant Capacity (TAOC) ELISA Kit	JLGR-E60168	0.5-50 U/mL	0.1 U/mL
IgG	Chicken Immunoglobulin G (IgG) ELISA Kit	JLGR-E60107	75-2400 μg/mL	10 μg/mL
IgM	Chicken Immunoglobulin M (IgM) ELISA Kit	JLGR-E60110	25-800 μg/mL	1.0 μg/mL
IgA	Chicken Immunoglobulin A (IgA) ELISA Kit	JLGR-E60105	10-320 μg/mL	1.0 μg/mL
LH	Chicken Luteinizing Hormone (LH) ELISA Kit	JLGR-E60197	0.312-78 pg/mL	0.1 pg/mL
FSH	Chicken Follicle Stimulating Hormone (FSH) ELISA Kit	JLGR-E60058	0.625-20 mIU/mL	0.1 mIU/mL
E2	Chicken Estradiol (E2) ELISA Kit	JLGR-E60056	15-480 pg/mL	1.0 pg/mL

Note: The intra- and inter-assay coefficients of variation (CV) for all ELISA kits were below 9% and 11%, respectively, as specified by the manufacturer.
